# One Health/EcoHealth capacity building programs in South and South East Asia: a mixed method rapid systematic review

**DOI:** 10.1186/s12960-017-0246-8

**Published:** 2017-09-29

**Authors:** Pranab Chatterjee, Abhimanyu Singh Chauhan, Jessy Joseph, Manish Kakkar

**Affiliations:** Public Health Foundation of India, Plot 47, Sector 44, Institutional Area, Gurgaon, 122 002 India

**Keywords:** One Health, EcoHealth, Capacity building, Research capacity, South Asia, South East Asia, Infectious diseases, Emerging infections, Zoonoses

## Abstract

**Background:**

Although One Health (OH) or EcoHealth (EH) have been acknowledged to provide comprehensive and holistic approaches to study complex problems, like zoonoses and emerging infectious diseases, there remains multiple challenges in implementing them in a problem-solving paradigm. One of the most commonly encountered barriers, especially in low- and middle-income countries, is limited capacity to undertake OH/EH inquiries. A rapid review was undertaken to conduct a situation analysis of the existing OH/EH capacity building programs, with a focused analysis of those programs with extensive OH engagement, to help map the current efforts in this area.

**Methods:**

A listing of the OH/EH projects/initiatives implemented in South Asia (SA) and South East Asia (SEA) was done, followed by analysis of documents related to the projects, available from peer-reviewed or grey literature sources. Quantitative data was extracted using a data extraction format, and a free listing of qualitative themes was undertaken.

**Results:**

In SEA, 13 unique OH/EH projects, with 37 capacity building programs, were identified. In contrast, in SA, the numbers were 8 and 11 respectively. In SA, programs were oriented to develop careers in program management, whereas, in SEA, the emphasis was on research. Two thirds of the programs in SEA had extensive OH engagement, whereas only one third of those in SA did. The target for the SEA programs was wider, including a population more representative of OH stakes. SEA program themes reveal utilization of multiple approaches, usually in shorter terms, and are growing towards integration with the traditional curricula. Such convergence of themes was lacking in SA programs. In both regions, the programs were driven by external donor agencies, with minimal local buy-in.

**Conclusions:**

There is limited investment in research capacity building in both SA and SEA. The situation appears to be more stark in SA, whilst SEA has been able to use the systematic investment and support to develop the OH/EH agenda and strategize capacity building in the core competencies. In order to effectively address the disease emergence hotspots in these regions, there needs to be strategic funding decisions targeting capacity building in the core OH/EH competencies especially related to transdisciplinarity, systems thinking, and adaptive management.

**Electronic supplementary material:**

The online version of this article (10.1186/s12960-017-0246-8) contains supplementary material, which is available to authorized users.

## Background

One Health (OH) or EcoHealth (EH) approaches have widely been considered to provide the most comprehensive and effective modes of managing the emerging infectious disease (EID) threats [1, 2]. However, despite the overwhelming consensus that the OH/EH approach needs to be deployed on a larger scale, there have been multiple challenges in implementing them. Developing an integrated and concerted response to EID challenges has been difficult, especially in times of crises, as it has been impeded by the lack of a prepared workforce [3].

Efforts like the tripartite agreement between the World Health Organization (WHO), Office International des Epizooties (OIE), and Food and Agriculture Organization (FAO) have strongly advocated for developing an OH workforce. A similar declaration in support of developing OH capacity, in addition to injecting innovative funding mechanisms to sustain research collaborations, was outlined by the American Veterinary Medical Association (AVMA) [4]. The European Union (EU), in a preparatory study to identify common bases for implementation of the OH approach in Europe and Asia, had identified OH capacity building to be a critical part of the process [5]. At the Stone Mountain Meeting on operationalization of OH, a special work group was created to focus on OH capacity building, with the mandate to raise awareness and expand engagement in the OH approach by leveraging existing programs and capacity building efforts [6]. A systematic review demonstrated the deficiencies in both OH capacity- and OH approach-based programs in the Indian context [7].

Though there have been multiple calls for capacity building in OH/EH competencies, and a slew of programs have been implemented over the last three decades, there has been a reticence about what needs to be done organizationally and policy-wise, in order to break out of sectoral interests and develop truly trans-sectoral training programs [8]. Thus, the need to develop an OH workforce, equipped with core set of competencies, was the result of the focus on the process, rather than the outcome [9]. The call to move away from the reductionist approaches and instead focus on cross-sectoral competency building through institutionalized and structured One Health capacity building has been growing louder [10].

Whilst the OH movement has gained a lot of momentum in the developed world, especially in North America, a concerted response has remained elusive in the developing regions [11–14]. Although several adaptations to accommodate the OH principles in existing capacity building initiatives have been undertaken, there has been limited success in converging the concepts of OH with the traditional training courses pursued by medical doctors, veterinarians, and other stakeholders in the OH movement [15]. For example, OH courses in the universities of developed countries have usually been restricted to the veterinarians, with limited involvement of other disciplines [16].

Studies have repeatedly shown that South and South East Asia are not only hotbeds for endemic zoonoses, but also hotspots for EIDs [17, 18]. With some of the most populous countries located in this region experiencing urbanization and economic stability, there has been a rapid expansion of the human-animal-environment interface. This, coupled with the rise of the intensive agricultural practices to meet the growing food security needs of the rapidly expanding population, has led to the emergence of potential vulnerabilities, which may manifest as emerging or re-emerging infectious disease threats [19]. Given these vulnerabilities, OH/EH capacity assumes immense significance as these regions prepare to respond to the EIDs and other OH challenges.

With this context in mind, it is essential to scrutinize the OH/EH capacity building (OHEHCB) efforts in the South Asia (SA) and South East Asia (SEA) regions. This was expected to help map the current efforts in this area. A thematic analysis of the programs would help to identify their features and make a bi-regional comparison to identify the gaps in the current efforts and thus help inform future endeavours in OHEHCB in the regions.

## Methods

A rapid systematic review was undertaken to conduct a situation analysis of the OHEHCB programs, with a focused analysis of those programs which have extensive OH engagement. This approach was preferred in order to streamline the process of building an informed, multi-stakeholder platform to enhance OHEHCB in the region. This review was conducted in order to facilitate the discussions of the platform and to help the members develop a roadmap for addressing the identified lacunae through concerted advocacy and planning. The expedited approach was favoured since we intended to provide the stakeholders with contextualized evidence that systematically addressed the objectives [20, 21]. We defined a project or an initiative to be the primary effort that received funding from the supporting agencies; OHEHCB programs were efforts directed at capacity building started under the aegis of an initiative or project. There could be multiple OHEHCB programs within one project/initiative.

The beginning point of the rapid review was a list of OH/EH projects or initiatives that were functional in the SA or SEA regions. To begin with, the mapping of these projects was done without a preference for a time bracket. A literature search was conducted using PubMed to identify papers which mentioned “One Health” or “EcoHealth” or “Ecosystems approaches to health” or “transdisciplinarity” in the title or abstract (limit [tiab]). Using the output, a list of projects was curated. Based on the results of this non-structured search, we then looked up the websites of the major funding agencies to identify further projects that qualified for inclusion in the review. This list was then iteratively screened by experts led by MK, to curate a final list of operational or concluded OH/EH projects in the SA and SEA regions.

Following this, a systematic search was conducted to identify documents related to the identified projects/initiatives to isolate information on capacity building functions included therein. This search was conducted to include peer-reviewed published literature (search was conducted in PubMed/MEDLINE, Cochrane Database of Systematic Reviews (CDSR), and IndMed); reports, articles, press releases, presentations, videos, and policy briefs published and indexed on CGSpace, the repository of agricultural research outputs hosted by the Consultative Group for International Agricultural Research (CGIAR); reports or updates on projects/initiatives obtained from the project websites; and unpublished/gray literature, including regulatory documents sourced through personal communications. We also undertook a manual search of the citations/references mentioned in the documents obtained from these sources to further increase the breadth of the included evidence. Further, to account for documents that may reflect on OHEHCB independently from the identified projects/initiatives, we searched the repositories/databases using structured search strategies.

We included any document which discussed the capacity building aspects of OH or EH projects or initiatives that were primarily conducted in countries belonging to the SA and SEA regions. Given the paucity of peer-reviewed publications, we chose a liberal inclusion criterion to include the maximum possible number of documents to review. Detailed inclusion/exclusion criteria are available in Technical Appendix 1 in Additional file 1.

The documents were initially included based on a review of the title and abstract in the case of peer-reviewed articles; in the case of the other documents, we included articles after reading the content and assessing to see if they fulfilled the inclusion criterion. Duplicates were removed using a reference management software. To expedite the process, the articles were screened by a single reviewer (PC), though the results were validated through a consultative, detailed presentation of the findings at a stakeholder meeting. This meeting had a wide variety of sectoral expertise, with experts hailing from medical sciences, veterinary sciences, public health, health policy, social sciences, and communication. Details of the experts are provided in Technical Appendix 3 in Additional file 1.

A data extraction form was created based on a pilot of three index documents (one research presentation, one report on a university network, and one project report on capacity building). The data extraction form was reviewed by another reviewer (MK) independently. This form extracted information on the particular capacity building programs deployed under the aegis of a project/initiative, the size of the programs, the countries (and region) in which they were deployed, the setting in which they were primarily organized, the duration of the programs, their primary functions and target audience, the extent of OH engagement undertaken in the program, and the major funding agency that supported the project/initiative under which the capacity building program was incorporated. Additionally, key themes about the programs, relating to the vision, mission, objectives, strategies adopted (including competency mapping), activities undertaken (including training programs and capacity building courses), and identified challenges/strengths, were also listed for a qualitative synthesis. Data extraction was done to the maximum extent of available information. Initially, we wanted to evaluate whether the identified programs adequately addressed the seven core competencies outlined by the South East Asia One Health University Network (SEAOHUN). These competencies include Collaboration and Partnership; Communication and Informatics; Culture, Beliefs, Values, and Ethics; Leadership; Management; Policy, Advocacy, and Regulation; and Systems Thinking [22]. However, early on in the reviewing process, we realized that most programs did not have adequate documentation to allow such an evaluation. So, programs which embraced transdisciplinary collaboration, systems thinking, and any three of the other elements were adjudicated by a reviewer (ASC) to have extensive OH engagement. Programs, which addressed at least transdisciplinarity and/or concepts of cross-sectoral collaboration and were adopted to meet the local needs (e.g. COHEART (the Center for One Health Education, Advocacy, Research and Training)), were considered to have locally adapted OH engagement. Those which failed to endorse any of these criteria completely in line with the SEAOHUN definitions and were oriented to training in a narrower skill zone were considered to have minimal OH engagement (e.g. the Joint Orientation Workshop on Zoonotic Diseases, India). When there was a conflict in decisions, an expert reviewer (MK) was consulted. The categorization of the programs based on these limited criteria is likely to over-estimate the number of programs with extensive OH engagement.

For the situation analysis and focused review, a descriptive-comparative approach was adopted; the focused review also helped identify best practice models and policies adopted by programs with the maximum extent of OH/EH engagement. For the qualitative review, a free listing of themes was done, followed by identification of recurrent themes, leading to the development of a conceptual framework, through an expert consultation at the stakeholder meeting. All major comparisons were made on a bi-regional basis to compare the pattern of OHEHCB programs in the SA versus the SEA region.

## Results

**Fig. 1:**
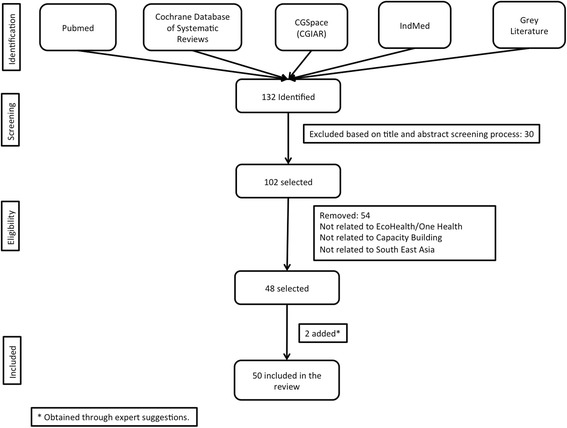
Flowchart showing details of inclusion of documents

Initially, 132 documents were retrieved from the various sources, of which 30 were not relevant to the research question. The remaining 102 documents were screened for relevance, in the course of which 54 were excluded as they did not satisfy the inclusion criteria. This left 48 documents, to which we added two articles which were obtained through expert suggestions; this brought the total number of papers included in the review to 50. The process is summarized in Fig. 1.

### Situation analysis

**Fig. 2:**
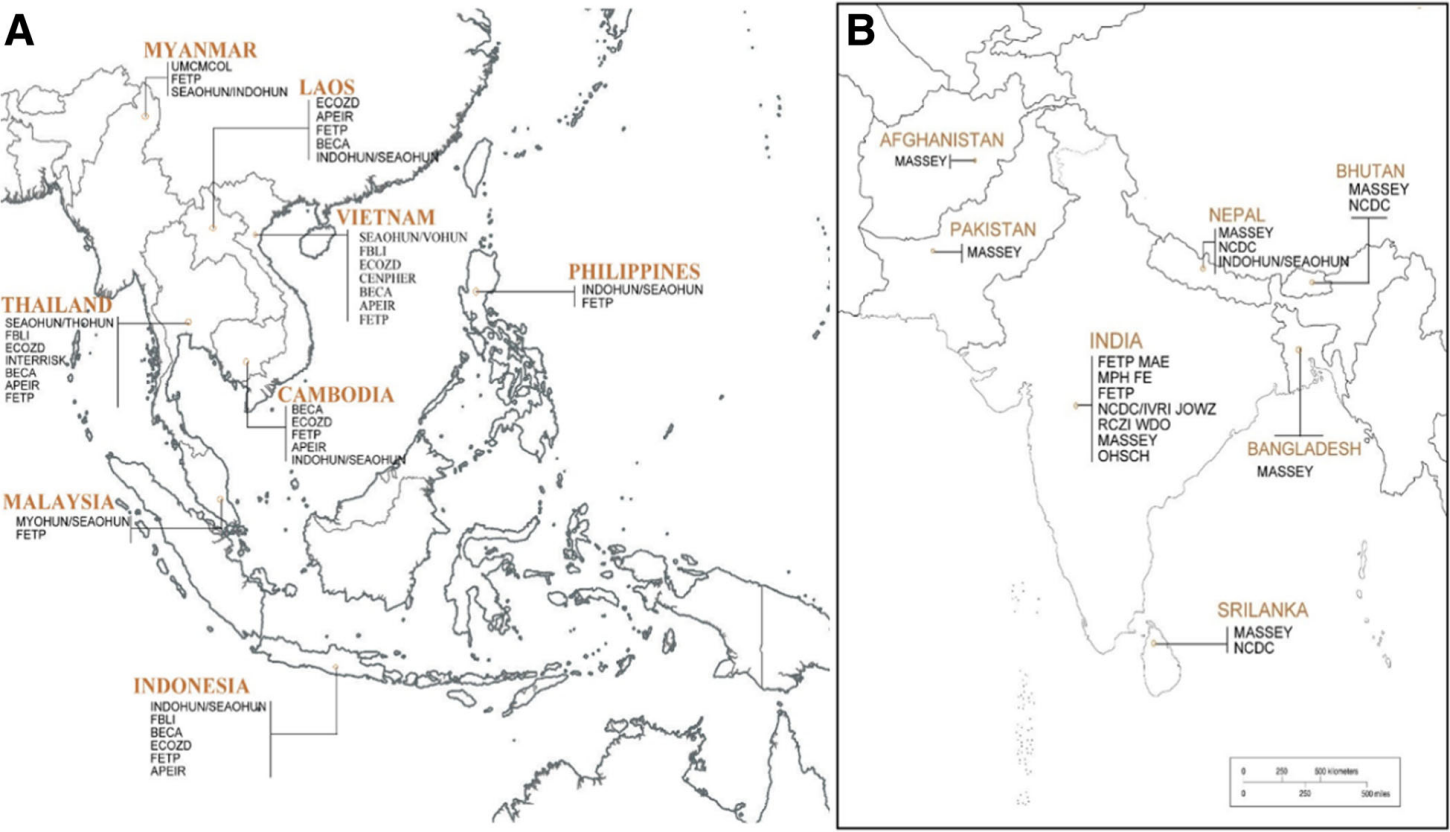
Map of all projects with One Health/EcoHealth capacity building components in South East Asia (**a**) and South Asia (**b**)

There were 13 unique OH/EH projects or initiatives which included 37 OHEHCB programs that were operational in countries of SEA. In SA, by contrast, this number was much smaller, with 8 projects/initiatives and 11 OHEHCB programs (Fig. 2; details in Technical Appendix 2 in Additional file 1).

Based on whether the programs were primarily focused on developing skills for surveillance, prevention, and control (e.g. Field Epidemiology Training Programs (FETPs)) or primarily focused on developing knowledge, research, and policy skills (e.g. Master’s programs), they were classified to be field setting-oriented or university setting-oriented programs. In both regions, more programs were university oriented; however, a larger proportion of programs had a field-level focus in SA (5/11; 45%) than in SEA (13/37; 35%). More programs in SA focused on developing professionals for a career in programs (8/11; 73%) rather than in research; in SEA, the opposite trend was seen, with more programs focusing on building research skills (25/37; 68%). There were differences in the duration of training as well. More programs in SA were of longer duration, lasting from months to years (6/11; 55%), than those in SEA (12/37; 32%). A large proportion of the OHEHCB programs in SEA were of shorter duration, lasting from days to weeks (22/37; 59%).

Based on the extent to which the OHEHCB programs addressed the core competencies outlined above, they were classified to have extensive engagement, locally adapted engagement, or minimal engagement [22]. A large proportion of the programs in SEA had extensive OH involvement (25/37; 68%). In contrast, only over a third of the programs based in SA had extensive OH engagement (4/11; 36%).

The programs in SA primarily targeted program managers (8/11; 73%) and veterinarians (2/11; 18%), whereas those in SEA primarily targeted a more representative spectrum of OH functionaries, including graduate students (14/37; 38%), researchers (11/37; 30%), program managers (6/37; 16%), medical students (3/37; 8%), and veterinarians (2/37; 5%).

### Focused review

There were more projects/initiatives with extensive OH engagement in SEA. In SA, there were only five OHEHCB programs in two projects/initiatives, whilst in SEA, there were 11 projects/initiatives with 25 OHEHCB programs with extensive OH engagement. Of the five programs in SA, four were based out of a university setting, whilst a little over half the programs in SEA were university based (14/25; 56%). Over half the programs in SEA were short term (13/25; 52%), whereas three of the five programs in SA were long term. We did not create any cut-offs to define short-, medium-, and long-term programs as we felt that such restrictions would impose artificial and illogical definitions. Short-term programs were organized for days to weeks and were meant for building a small set of skills; these programs, often conducted in a workshop mode, usually did not culminate in the acquisition of any degrees or certification. Medium-term programs lasted for weeks to months and were meant primarily for skill enhancement of public health professionals, program managers, and in-service candidates; they may have resulted in certification but not the acquisition of a degree. Long-term programs were carried on for months to years, usually in a university setting, and cumulated in the acquisition of a degree or certificate subject to multiple assessments over the period of the coursework.

Three of the five programs in SA focused on developing professionals for a career in programs rather than research and targeted program managers. In contrast, a majority of the programs in SEA (16/25; 64%) were focused on building research skills, and the programs were targeted to reach a wide spectrum of potential OH workforce members, including graduate students (10/25: 40%), researchers (7/25; 28%), medical students and program managers (3/25 each; 12%), and veterinarians (2/25; 8%). Table 1 shows the spread of programs across the SA and SEA regions, based on their duration and primary function. Although we defined the extent of adherence to the principles of OH and EH to identify documents for the focused review, given the qualitative nature of the enquiry, and the potential gap between documented protocols and the way they were deployed in the challenge of real-world settings, there is a possibility that we may have over-estimated the number of programs in this section of the review.

**Table 1:** Duration and function of programs with extensive OH engagement by region

Region	Duration	Functions	
		Program	Research
SA	Long term (months to years)	2	2
	Short term (days to weeks)	1	0
SEA	Long term (months to years)	0	8
	Medium term (weeks to months)	0	2
	Short term (days to weeks)	8	5

### Thematic analysis

On analysing the listed themes, it was observed that most of the programs in SEA were driven by the OH approach, whereas those in SA had an FETP-oriented, disease control-based approach, with limited emphasis on OH concepts. The divergence of the approach to OH capacity building in the programs in the two regions is summarized in Table 2. However, as has been pointed out earlier, adequate documentation to assess the curricular extent of each of the programs was not available. In light of this experience, we decided to use a less stringent approach to define the extent of OH engagement. Despite that, we found a small fraction of the programs to fulfill the set criteria. The current data suffers from the uncertainty spawned by the inadequacy of the available documentation.

**Table 2:** Divergence in the approach to OH capacity building programs in SEA and SA

South East Asia	South Asia
One Health approach predominant	FETP-oriented, disease control-based approach with limited emphasis on One Health concepts
Networks present; supportive frameworks within countries and across borders	No indigenous networks present with focus on OH capacity building; One Health Hubs, created in project-mode initiatives, were the hallmark of some of the programs with extensive OH engagement.
Capacity building is driven by a competency-based approach	Outcome-oriented approach—with focus on surveillance and response to disease outbreaks and limited emphasis on other competencies; OHEHCB efforts under one initiative focused on implementing collaborative, investigation projects as part of the training package.
Most commonly adopted curricular model was one based on core competencies and technical competencies.	Topic-based curricula followed for most OHEHCB programs; except for one program, emphasis on competencies-based approach has been limited.

There were fundamental differences in the strategies that were deployed in order to build the capacity of the present and future OH workforce between programs in each region. In some countries, like Thailand, there were curricular modifications based on the target group. As an example, duration and field experience levels were shorter and more intense for in-service program managers, whereas for graduate students and other full time participants, the students were expected to work towards a degree or certification. In countries like Vietnam, there were initiatives to integrate OH modules into the regular curricula for medical and veterinary students. The divergence in the strategies are summarized in Table 3. Since the data for the thematic review was sourced from multiple types of documents, which were not screened for quality, we anticipate that there might be some over-estimation of the potential of the identified OHEHCB efforts to adhere to the tenets of OH and EH.

**Table 3:** Divergence in the strategies for OH/EH capacity building in SEA and SA

South East Asia	South Asia
• Multiple approaches for fulfilling the needs of different target audience groups • Degree/certificate courses for graduate students generally of longer duration • Shorter terms of training preferred for more experienced target groups, especially for program managers • Integration of OH modules with regular medical and veterinary courses	• Focused on developing disease surveillance and outbreak reporting skills with limited focus on other competencies • Almost all courses culminate with the students getting a degree or certification of skills • OH concepts are acknowledged but largely unaddressed in the curricula • Generally driven by sectoral interests and cross-sectoral teaching-learning is minimal

## Discussion

The paucity of peer-reviewed literature documenting OHEHCB efforts in the two regions was an early indicator that there has not been an adequate focus on it. OHEHCB initiatives in both the regions are a recent phenomenon and could reflect the consequence of repeated EID events that have incited the interest of donors and the international community at large. Except for some country-to-country variations, the agenda have essentially been moved by international donor agencies, with ownership by national governments still emerging. OHEHCB programs across the regions have been characterized by universities as an entry point for initiating the movement. This is similar to the North American experience where the OHCB movement has traditionally been led by schools of veterinary medicine and public health in different universities. Common focus on both regions on field epidemiology as an essential component of OHEHCB is an indication of the ever burgeoning need to strengthen the frontline capacity of health systems. Additionally, in both the regions, capacity building in wildlife-related issues was limited. Considering the fact that almost three fourths of emerging zoonoses originate from wildlife sources [23], this is a major area of concern that needs to be addressed in future OHEHCB efforts.

From the identified initiatives, it was observed that the major support was obtained through the International Development Research Centre (IDRC), Canada, and the United States Agency for International Development (USAID). The Avian and Human Influenza Fund (AHIF), via the World Bank and the European Union, have also funded two significant OHEHCB programs in the region. In both the regions, the relative absence of buy-in from the local research and funding agencies, including universities, raises questions about the sustainability of the efforts. In SEA, some indigenous programs are in the initial phases of conceptualization. This is an encouraging finding since without support from local agencies, long-term sustainability of OHEHCB efforts would be questionable.

Several differences were found to exist in the overall OHEHCB strategy between the two regions; it is possible that this could be critical in determining the overall preparedness to EID events, not only for the regions overall, but also for the individual member states that are located in the respective regions. Overall, there has been a stronger response to OHEHCB in SEA as compared to SA. Not only were there more projects or initiatives committed to build capacity in OH/EH in the SEA region, but there were more capacity building programs within each of these projects. Further, there was a more systematic focus on building OHEHCB in SEA; there are two regional EcoHealth Research Centres (EHRCs), at Chiang Mai University (CMU), Thailand, and Universitas Gadjah Mada (UGM), Indonesia. Additionally, there is a Centre for Public Health and Ecosystem Research (CENPHER) housed at the Hanoi School of Public Health, Vietnam. There were no comparable initiatives in SA. The OHEHCB endeavours across the various SEA countries were unified under the activities of the South East Asia One Health University Network (SEAOHUN). In addition to providing curricular guidance, the network also supports member countries in building capacity to respond to OH policy needs. Whilst such policy structures are absent in SA, an OH policy for response to emerging infectious disease threats has been outlined in Bangladesh. Although this is not comparative to the policy mandate adopted by the SEAOHUN, a policy-level commitment to deploying OH interventions for EID threats could set the tone for structured OHEHCB programs in the region [24]. The current review happens to mirror the findings of a previous review by Hung et al., in which they found a multitude of projects and initiatives in OH/EH which were supported by major international donors [25]. Although a number of projects and initiatives have funded capacity building efforts directed at the stakeholders, there has been limited uptake of the same in the national policies across the nations in both the regions. Aside from a small program in Vietnam, integration of OHEHCB with medical and veterinary curricula remains a theoretical construct for most nations. This reluctance on behalf of national players to commit to developing core OHCC in stakeholders is reflective of the ambivalent approaches to the policymaking discourse in developing nations, in which OH remains at an arm’s length [26]. There has been much debate about what should comprise the ideal mix of competencies, but that discussion is beyond the scope of the current review and we have limited ourselves to the defined competencies outlined by the SEAOHUN in the context of vulnerable settings in developing or low- and middle-income countries [27].

Another key difference was observed: whilst the programs in SA were oriented to developing program managers with the skills for disease surveillance and outbreak investigation, the programs in SEA, additionally, focused on providing the OHCCs to the trainees in addition to technical competencies. With the exception of the OHEHCB initiatives under the leadership of Massey University, the programs in the SA region were largely geared towards fulfilling programmatic needs rather than addressing research and development capacity issues [28]. Whilst there were too few initiatives in SA that had extensive OH engagement for the trainees, the overwhelming focus on programmatic needs is a reflection of the perceived priorities of the region, where disease reporting and response systems remain weak. The complementarity seen in the programs in SEA could be a reflection of the fact that the SEAOHUN, the major network driving recent capacity building efforts in the region, based its activities on a set of core competencies and technical competencies, which addressed both types of skills.

In addition, the review findings also indicate limited commitment to the use of systematically developed criteria or standards to assess whether the framed curricula actually address the One Health competencies that they intend to develop capacity in. This is further complicated by the fact that aside from the SEAOHUN, there have been very limited efforts in SA or SEA to develop an evidence-based competency matrix on which to frame the scaffolding of OHEHCB programs. A recent publication on the operational criteria for ecosystem approaches in health proposes the sequential integration of skills related to transdisciplinarity, systems thinking, and adaptive management [29]. However, none of the programs we identified in the SA region used such a framework, a priori, to establish a structured OH/EH approach to capacity building in key stakeholders.

Although the terms multidisciplinary, interdisciplinary, and transdisciplinary are used interchangeably, they represent very different concepts. Most of the OH programs in the SA region that claimed to have a OH component were seen to be limited to functioning within the multidisciplinary framework—the system where researchers from different fields work sequentially or in parallel, but independently, and within their disciplinary perspectives [30]. In SA, the more recent programs deployed by Massey University represent the sole example where collaborative investigation project work was taken up till the policymaking level, achieving some success in interdisciplinary cooperation. Yet, given that a larger proportion of programs in SEA focussed on the core OH competencies, it is more likely that they have moved further along the continuum towards achieving truly transdisciplinary training of their students/trainees—one where researchers, program managers, students, community members, and policymakers come together to work with a shared vision, drawing together knowledge from scientific, social, economic, and other relevant contexts, to devise a comprehensive solution for a complex, cross-cutting problem, like EIDs [31]. This remains a concern, especially in the light of the lack of structured framework to address the capacity gaps. Given the *ad hoc*ism noted in several programs of OHEHCB in both the regions, but more so in SA, the challenge remains to build programs with evidence-driven identification of core competencies and structured curricula developed to hone these competencies, involving a wider cadre of beneficiaries, including stakeholders who may be affected by the OH/EH policies. In addition, there is a dire need to assess the extent to which OHEHCB programs are actually leading to capacity enhancement in target groups; for this, there may be a need to develop new standards or adapt existing ones to fit the local context [29].

In the main review, we chose to exclude some of the ongoing or recently concluded OH/EH initiatives, especially since a more complete understanding of their impact is forthcoming. However, these initiatives are too few in number to substantially alter the conclusions of the current review. They include four projects: Building Research Excellence in Wildlife and Human Health in Sri Lanka; Climatic Variability, Societal Changes, and Dengue Disease in Bangladesh; Addressing Bovine Tuberculosis and Veterinary use of Antibiotics in Smallholder Peri-urban Dairy Farms in India; and Reducing Biosecurity Threats from Infectious Diseases of Pandemic Potential in Southeast Asia. These studies have incorporated a capacity building component; however, more results need to be published before their impact can be fully assessed.

The current review has several limitations. Owing to the rapid review approach adopted, there has been a limited extent and depth to which the documents were sourced from. Almost none of the programs had their curricula available on the public domain, making it difficult to appreciate the finer nuances of these programs. However, it provides a summary outline of the ongoing efforts, opens up channels of communication, and helps with securing evidence to develop a strategic plan.

## Conclusion

In spite of the relevance of OHEHCB in both the regions, the response has been generally slow. SEA has made more progress compared to SA in terms of quantum, quality, and relevance. However, the movement has essentially been externally driven with minimal institutionalization in the form of policy support and ownership by the governments. This questions the sustainability of the efforts. Several intra-country and inter-country collaborative mechanisms created in the aftermath of the avian influenza outbreaks have not only brought together national resources but also galvanized political opinion around the need for intersectoral action. These platforms are an ideal framework on which to build the OHEHCB movement.

## Additional file

Additional file 1: Technical Appendix 1. Inclusion and exclusion criteria. **Technical Appendix 2.** One Health/EcoHealth programs and capacity building programs included in the review. **Technical Appendix 3.** details of experts in stakeholder meeting.

## Abbreviations

AVMA: American Veterinary Medical Association
CDSR: Cochrane Database of Systematic Reviews
CENPHER: Center for Public Health and Ecosystem Research, Vietnam
CGIAR: Consultative Group for International Agricultural Research
CMU: Chiang Mai University, Thailand
EH: EcoHealth
EHRCs: EcoHealth Research Centers
EID: Emerging Infectious Diseases
EU: The European Union
FAO: Food and Agriculture Organization
FETPs: Field Epidemiology Training Programs
IDRC: International Development Research Center
OH: One Health
OHCC: One Health Core Competency
OHEHCB: OH/EH capacity building
OIE: Office International des Epizooties; World Organisation for Animal Health
SA: South Asia
SEA: South East Asia
SEAOHUN: South East Asia One Health University Network
UGM: Universitas Gadjah Mada, Indonesia
USAID: United States Agency for International Development
WHO: World Health Organization
