# Supplementary material for: One Health/EcoHealth capacity building programs in South and South East Asia: a mixed method rapid systematic review
Source: Hum Resour Health. 2017 Sep 29;15:72. doi: 10.1186/s12960-017-0246-8 (PMC5622563; doi:10.1186/s12960-017-0246-8)
Supplement: Additional file 1: Technical Appendix 1. — Inclusion and exclusion criteria. Technical Appendix 2. One Health/EcoHealth programs and capacity building programs included in the review. Technical Appendix 3. details of experts in stakeholder meeting. [file 12960_2017_246_MOESM1_ESM.docx]

# Technical Appendix 1: Inclusion and Exclusion Criteria

## Inclusion Criteria

**Types of documents:**

1. Peer reviewed articles: qualitative and quantitative studies (including mixed methods studies), reviews (systematic or otherwise), opinion pieces, editorials, personal perspectives, case studies, etc.
2. Reports, including regulatory submissions to funding agencies
3. Articles in non-peer reviewed publications (e.g. newsletters)
4. Press releases
5. Presentations and slideshows
6. Policy briefs
7. Project updates available in the public domain online
8. Regulatory documentations, including those obtained through personal communication

**Types of Subjects:**

1. Projects or initiatives, which were primarily supported by funding agencies through grants and which had One Health Capacity Building as one of the core objectives, or which had a declared focus on this.
2. Any particular program, related to One Health/EcoHealth Capacity Building, launched under the aegis of a larger project or initiative (which received grant support).

**Regions:**

1. South Asia, represented by Afghanistan, Pakistan, India, Nepal, Bhutan, Bangladesh, Maldives, and Sri Lanka. Based on the membership of the South Asian Association for Regional Co-operation (SAARC).
2. South East Asia, represented by Myanmar, PDR of Lao, Viet Nam, Philippines, Cambodia, Thailand, Malaysia, Indonesia, Brunei Darussalam and Singapore. Based on the membership of the Association of South East Asian Nations (ASEAN).

## Exclusion Criteria

1. Any country which falls within the region but has no One Health or EcoHealth capacity building project implemented in it were excluded from the analysis.
   - No programs could be mapped to Maldives, except sporadic participation in NCDC India’s FETP, hence it was excluded. No programs could be mapped to Maldives, except sporadic participation in NCDC India’s FETP, hence it was excluded. Similarly, no programs could be mapped to Singapore and Brunei Darussalam, which were excluded from the analysis.
2. Ongoing projects or recently concluded projects with limited documentation were included in a separate segment and were not a part of the main analysis.

# Technical Appendix 2: One Health/EcoHealth Programs and Capacity Building Programs included in the review

| **OH/EH Projects/Initiative** | **OHEH Capacity Building Programs** | **Implementing Agencies/Bodies** |
| --- | --- | --- |
| South East Asia One Health University Network (SEAOHUN) | - | 1. Institut Pertanian Bogor  2. Universitas Gadjah Mada  3. Universitas Indonesia  4. Universiti Kebangsaan Malaysia  5. Universiti Putra Malaysia  6. Chiang Mai University  7. Mahidol University  8. Hanoi Medical University  9. Hanoi School of Public Health  10. Hanoi University of Agriculture  11. Tufts University  12. University of Minnesota |
| Thailand One Health University Network (THOHUN) | 1. THOHUN-Tufts environmental literacy course 2. OH Workforce Development in Thai University Network 3. Integrative OH Modules for Current and Future OHW 4. In class training course on principle and practice of OH 5. Short course on Rapid Response to Emerging Infectious Disease outbreaks | 1. Mahidol University  2. Chiang Mai University  3. Chulalongkorn University  4. Kasetsart University  5. Prince of Songkla University  6. Thammasat University  7. Mahasarakham University  8. Khon Kaen University |
| Vietnam One Health University Network (VOHUN) | 1. Field Epidemiology Short Course 2. OH Module (Elective) in Graduate Medical Education 3. Infectious/Zoonotic Disease Short Courses 4. Training of Trainers | 1. Vietnam National University of Agriculture  2. Hanoi University of Public Health  3. Institute of Preventive Medicine and Public Health  4. HCM University of Pharmacy and Medicine  5. Hue University of Agriculture and Forestry |
| Malaysia One Health University Network (MYOHUN) | 1. OH Diagnosis of Leptospirosis 2. OH Short Course 3. Workshop for Training of Communication and Leadership for One Health Community Education 4. Training of Trainers | 1. Universiti Putra Malaysia (UPM)  2. Universiti Kebangsaan Malaysia (UKM) |
| Indonesia One Health University Network (INDOHUN) | 1. Global Health True Leaders 2. Global Health True Leaders Training of Trainers 3. OH Training 4. OH Collaborative Internship Program | 1. Syriah Kuala University  2. University of North Sumatera  3. Andalas University  4. Brawijaya University  5. Udayana University  6. Mulawarman University  7. Sam Ratulangi University  8. Hassanudin University  9. Cendrawasih University  10. University of Nusa Cendana  11. University of Mataram  12. University of West Nusa Tenggara  13. Diponegoro University  14. Airlangga University  15. Gadjah Mada University  16. Padjadjaran University  17. Bogor Agricultural Institute  18. Universitas Indonesia  19. Sriwijaya University  20. Prof. Buya Hamka University |
| Building EcoHealth Capacity in Asia (BECA) | - | Veterinarians without Borders |
| Field Building Leadership Initiative | 1. Future Leaders Training in EcoHealth 2. Site-Based Research 3. Global Health Course with EcoHealth Module 4. Multidisciplinary EcoHealth Graduate Course 5. EcoHealth Degree Program 6. Introduction to EcoHealth 7. EcoHealth Electives    1. For undergraduates    2. For medical graduates 8. Training of Trainers | FBLI consortium:  1. Health Systems Research Institute, Ministry of Public Health, Thailand  2. Department of Environmental Health, Hanoi School of Public Health, Vietnam  3. Center of Excellence for Vectors and Vector-Borne Diseases, Faculty of Science, Mahidol University, Thailand 4. Institute for Health and Development Studies, Kunming Medical University, China  5. Kunming Institute of Botany, China  6. Faculty of Public Health, Universitas Indonesia  7. Veterinarians without Borders/Vétérinaires sans Frontières-Canada (VWB/VSF) |
| EcoHealth Emerging Infectious Disease Initiative (EcoEID) | 1. Research Based Capacity Building | 1. Centre for Malaria Control of the Government of Cambodia  2. Institute of Ecology and Biological Resources, Viet Nam  3. Jiangsu Institute of Parasitic Disease, China  4. National Institute of Public Health, laos  5. Public Health Foundation of India, India  6. Asian Foundation for Tropical Medicine, Inc., Philippines  7. Khon Kaen University, Thailand  8. Veterinarians Without Borders, Canada  9. China Agricultural University, China  10. Chinese Center for Disease Control and Prevention, China  11. Hanoi School of Public Health, Viet Nam  12. International Centre of Insect Physiology and Ecology, Kenya  13. Mahasarakham University, Thailand  14. Ubon Ratchathani University, Thailand  15. Vietnam Institute of Policy and Strategy in Agricultural and Rural Development, Viet Nam  16. Agence Tunisienne de Coopération Technique, Tunisia  17. Centre for Coastal Health Society, Canada  18. Mahidol University, Thailand  19. Research Institute for Tropical Medicine, Philippines  20. The Indonesian Center for Agriculture Socio-Economic and Policy Studies, Indonesia  21. Yaman Lahi Foundation, Inc. - Emilio Aguinaldo College, Philippines |
| Ecosystem Approaches to the Better Management of Zoonotic Emerging Infectious Diseases in South East Asia (EcoZD) | 1. Research Projects inculcating Learning by Doing 2. EcoZD-FBLI EcoHealth Short Course 3. EcoHealth Research Center activities | 1. International Livestock Research Institute  2. EHRC, Chian Mai University, Thailand  3. EHRC, Universitas Gadjah Mada, Indonesia  4. Six country partners |
| Center for Public Health and Ecosystem Research (CENPHER) | 1. Introduction to EcoHealth for Specialist, Master's and Bachelor students 2. One Health Risk Assessment Short Course | Hanoi University of Public Health |
| OH Master’s education and applied epidemiology training | One Health Regional Training in Animal and Human Health Epidemiology | Massey University |
| Master of Public Health (Biosecurity) and Master of Veterinary Medicine (Biosecurity) degrees | One Health Education in Epidemiology and Biosecurity | Massey University |
| OH Epidemiology Fellowship Program | Integrating Education and Action for One Health | Massey University |
| OH research and training activities | Veterinary Education Twinning Program | Massey University and University of Peradeniya, Sri Lanka |
| CMU-UMinn Collaboration | 1. University of Minnesota - Chiang Mai University Collaboration for Myanmar Initiative | Chian Mai University, Thailand |
| INTERRisk | One Health Master's Double Degree - Master's for assessment and management of health risks at the human, animal and ecosystem interface | 1. Kastesart Unviversity (Bangkok, Thailand)  2. Paul Sabatier University (Toulouse, France) |
| Regional and National Training Programs | 1. Field Epidemiology Training Program 2. Field Epidemiology Training Program Master's in Applied Epidemiology 3. Master's in Public Health Field Epidemiology | National Centre for Disease Control, India |
| Veterinary Public Health Capacity Building Program | 1. Joint Orientation Workshop on Zoonotic Infections | National Centre for Disease Control – Indian Veterinary Research Institute – World Health Organization |
| Training module for district level officers | 1. Workshop on Integrated Zoonotic Disease Prevention and Control | Roadmap to Combat Zoonoses in India Initiative, Public Health Foundation of India, India |
| Multiple nations in SEA through WHO | 1. International Short Course on Veterinary Field Epidemiology in Action | World Health Organization |
| Asia Partnership on Emerging Infectious Diseases Research (APEIR) | 1. Approaches using Knowledge Management and Collaboration for Capacity Building 2. Using the Workshop Approach to build research capacity | 1. Viet Nam: 13 institutions  2. Thailand: 10 institutions  3. Indonesia: 8 institutions  4. Cambodia: 4 institutions  5. China: 13 institutions  6. Lao PDR: 3 institutions |
| Summer School Program for Veterinary Students | 1. One Health International Summer School | Guru Angad Dev Veterinary School, India and Saskatchewan University, Canada |
| Center for One Health Education, Advocacy, Research and Training | 1. Postgraduate Certificate in One Health 2. Postgraduate Diploma in One Health | Kerala Veterinary Animal Science University |

# Technical Appendix 3: Details of Experts in Stakeholder Meeting

| **Sl. No.** | **Sectoral Expertise** | **Scope of Work** |
| --- | --- | --- |
| 1 | Community Medicine, Health Policy | National |
| 2 | Public Health | National |
| 3 | Public Health | International |
| 4 | Veterinary Public Health | National |
| 5 | Infectious Diseases | National |
| 6 | Wildlife Veterinarian | National |
| 7 | Funding Agency, Health Policy, One Health/EcoHealth | International |
| 8 | Veterinary Public Health | International |
| 9 | Communication | National |
| 10 | Veterinary Public Health, Food Safety | National |
| 11 | Social Sciences | Local |
| 12 | Communication | Local |
| 13 | Infectious Diseases, Health Policy, One Health/EcoHealth | International |
| 14 | Epidemiology, Public Health | National |
| 15 | Social Sciences | National |
| 16 | Capacity Building, Public Health | National |
| 17 | Capacity Building, Veterinary Public Health, One Health/EcoHealth | Regional |
| 18 | Capacity Building, Veterinary Public Health | International |
| 19 | Veterinary, Health Policy | National |
